# Passion, Motivation, and Well-Being in Young Footballers: A Systematic Review

**DOI:** 10.3390/healthcare13243273

**Published:** 2025-12-13

**Authors:** Diogo Braz, Cátia Maia, Élvio Gouveia, Diogo Monteiro, Nuno Couto, Hugo Sarmento

**Affiliations:** 1 University of Coimbra, Interdisciplinary Center for the Study of Human Performance (CIPER), Faculty of Sport Science and Physical Education, 3040-248 Coimbra, Portugal; diogosalgadobraz@gmail.com (D.B.); catiaarmaia@gmail.com (C.M.); hugo.sarmento@uc.pt (H.S.); 2 Department of Physical Education and Sport, University of Madeira, 9020-105 Funchal, Portugal; erubiog@staff.uma.pt; 3 Laboratory of Robotics and Engineering Systems, Interactive Technologies Institute, 9020-105 Funchal, Portugal; 4 Research Center in Sport Sciences, Health Sciences and Human Development (CIDES), 5001-801 Vila Real, Portugal; diogo.monteiro@ipleiria.pt; 5 ESECS, Polytechnic University of Leiria, 2411-901 Leiria, Portugal; 6 Sport Sciences School of Rio Maior, Santarém Polytechnic University (ESDRM-IPSantarem), 2040-413 Rio Maior, Portugal; 7 CIPER, Faculdade de Motricidade Humana, Universidade de Lisboa, Cruz-Quebrada-Dafundo, 1649-004 Lisboa, Portugal

**Keywords:** harmonious passion, obsessive passion, self-determination theory, youth football players, basic psychological needs, well-being in sports

## Abstract

**Highlights:**

**What are the main findings?**

- The review shows that harmonious passion in youth football players is positively linked to motivation, satisfaction of basic psychological needs, positive emotions, and overall life satisfaction. In contrast, obsessive passion is associated with negative outcomes, such as burnout and psychological distress. These results highlight the relevance of the Dualistic Model of Passion and Self-Determination Theory in understanding motivation and well-being among young athletes.

**What are the implications of the main findings?**

- Practically, the findings suggest that promoting harmonious passion and supporting athletes’ psychological needs can improve both performance and mental health in youth football. Future research should employ longitudinal designs and larger samples to further validate these relationships and help define effective strategies for coaches, parents, and sport organizations.

**Abstract:**

**Background:** Psychological well-being is crucial for the development and performance of young athletes. This systematic review aims to synthesize the available scientific evidence on the relationship between passion (harmonious and obsessive), basic psychological needs (BPNs), motivation, affect (positive and negative), and life satisfaction in young football (soccer) players. **Methods:** A systematic literature review was performed, following the PRISMA 2020 (Preferred Reporting Items for Systematic Reviews and Meta-Analyses) guidelines. The search was conducted in the Web of Science, Scopus, ERIC, and SportDiscus databases, using a comprehensive strategy that combined keywords related to football, youth, passion, motivation, and well-being. Two independent reviewers performed article screening, eligibility assessment, and data extraction. The methodological quality of the included studies was determined using two different tools. **Results:** Nine studies met the inclusion criteria and were analyzed in detail. The results consistently indicate that harmonious passion is associated with greater fulfillment of BPNs, positive affect, and overall life satisfaction. In contrast, obsessive passion was linked to negative outcomes such as burnout and emotional dysregulation. The available evidence suggests a positive association of harmonious passion with motivation and well-being, and an association of obsessive passion with psychological distress. **Conclusions:** Within the delimited scope, the evidence suggests that harmonious passion is an important construct positively associated with the well-being and motivation of young footballers, while obsessive passion is associated with adverse outcomes. Research in this area is scarce, showing methodological diversity and heterogeneous samples, which limits the generalizability of the findings. Future research should prioritize longitudinal designs and interventions to promote harmonious passion and the satisfaction of BPNs.

## 1. Introduction

Football, the world’s most popular sport, extends beyond mere entertainment and competition. It enhances self-esteem, fosters social connections, and reduces stress and anxiety [1]. The sport can significantly impact players’ lives by promoting growth, motivation, and social interaction, affecting their psychological needs [2]. Soccer provides an ideal setting to explore how passion is experienced, as its complexity encourages personal growth and mastery [3] because its practical complexity evokes passion and cultivates an environment focused on personal growth and mastery [4]. Therefore, Passion for sports plays a fundamental role in the experience of young athletes, influencing not only their performance but also their well-being and life satisfaction [5,6]. The Dualistic Model of Passion (DMP) posits that passion can be classified into two distinct types: harmonious passion and obsessive passion [7]. Harmonious passion occurs when sports participation is autonomously and adaptively internalized, allowing the individual to engage in the activity without compromising other life domains [5]. Conversely, obsessive passion results from a controlled internalization process, leading to a rigid compulsion to engage in the activity, often causing conflicts between sports participation and other life spheres [5,8].

Empirical studies show that harmonious passion is positively associated with well-being indicators such as positive affect, intrinsic motivation, and life satisfaction, whereas obsessive passion is linked to negative emotions, stress, and burnout [9,10,11,12]. Furthermore, obsessive passion has been found to contribute to maladaptive outcomes such as emotional exhaustion and decreased motivation over time [13]. Research also indicates that obsessive passion may lead to increased susceptibility to injury due to overtraining and an inability to disengage from sport despite physical limitations [14].

The Self-Determination Theory [15,16,17] offers a framework for understanding motivation and its impact on athletes’ well-being. It posits a continuum of motivation, from amotivation (lack of intention) to extrinsic motivation (various forms of external regulation) and ultimately to intrinsic motivation (enjoyment of the activity), the latter being linked to greater engagement and satisfaction [15,16,17,18]. Within SDT, the Organismic Integration Theory (OIT) within SDT explains how external regulations can be internalized, shaping athletes’ behaviors and experiences [15]. Research shows that when young footballers autonomously internalize motivation, they demonstrate greater persistence, improved performance [19], and higher satisfaction with their sports participation [20]. In contrast, externally regulated motivation—due to pressure from parents or coaches—can lead to stress, anxiety, and dropout [21,22,23,24].

The Basic Psychological Needs Theory (BPNT), a sub-theory of SDT, posits that satisfying three key psychological needs, autonomy, competence, and relatedness, is vital for well-being and optimal performance in sports [15,16]. In youth football, autonomy involves athletes’ control over their sports choices, competence relates to their ability to perform tasks effectively, and relatedness reflects the quality of interactions with coaches, teammates, and family [25]. Empirical evidence supports that satisfying these needs enhances intrinsic motivation, fosters greater enjoyment, and contributes to better performance and psychological well-being [19,22,26,27]. When these needs are thwarted, athletes experience heightened stress, increased risk of burnout, and lower engagement levels [28,29].

The interplay between passion and SDT constructs has been widely examined in sports psychology literature (e.g., Ref. [12]). Studies show that harmonious passion is closely linked to self-determined motivation and the fulfillment of psychological needs, promoting well-being and positive affect [7,11,21,30]. Athletes with harmonious passion report higher levels of flow states, lower anxiety, and greater resilience in the face of challenges [31,32]. In contrast, obsessive passion relates to controlled motivation, which can lead to emotional distress, performance anxiety, and increased dropout rates [33]. The negative impact of obsessive passion on basic psychological needs has been noted in various sports, highlighting the risks of a compulsive training approach [34]. Well-being in sports can be assessed through three primary dimensions: affect, life satisfaction, and psychological functioning [35]. Affect involves the experience of emotions, while life satisfaction reflects an athlete’s overall well-being evaluation [36]. Research indicates that harmonious passion and self-determined motivation are positively associated with positive affect, higher life satisfaction, and adaptive coping strategies [15,37]. On the other hand, obsessive passion and controlled motivation are linked to negative affect, maladaptive perfectionism, and increased susceptibility to burnout [9].

Moreover, research underscores the importance of the sports environment in shaping young footballers’ experiences. Supportive coaching that promotes autonomy and competence enhances intrinsic motivation, while controlling styles lead to anxiety and distress [6,38]. Longitudinal studies show that an autonomy-supportive climate in youth football fosters sustained engagement and personal development [28,39]. Additionally, parental support and peer relationships are vital for young footballers’ sense of relatedness and psychological well-being [40].

The study is based on the Dualistic Model of Passion and Self-Determination Theory, which explores the relationships among passion (harmonious vs. obsessive), motivation, psychological need satisfaction, burnout, and performance in youth football. The Dualistic Model outlines the consequences of different types of passion, while Self-Determination Theory addresses how autonomy, competence, and relatedness impact athletes’ psychological well-being. Research indicates that harmonious passion enhances well-being, whereas obsessive passion often results in negative outcomes, particularly under external pressures. Understanding this interplay is crucial during the developmental stages of young athletes facing increasing competition.

By examining the relationship between passion, need satisfaction, motivation, well-being, and performance, this study shifts the focus from training quantity to motivation quality in sustaining sports participation. It also identifies a potential risk period of diminishing autonomous motivation among older academy athletes, offering both theoretical insights and practical recommendations for coaching environments that promote long-term development, mental health, and optimal performance in young football players. To address this gap, the present study is guided by the following research questions: What is the relationship between harmonious and obsessive passion, motivation, psychological need satisfaction, and well-being in youth football players? What is the relationship between these constructs and burnout, as well as performance outcomes? What are the implications of these relationships for fostering long-term engagement, mental health, and optimal development in high-performance youth football? By explicitly framing the study around these questions, the research provides a clear and systematic basis for investigating the interplay between passion, motivation, and psychological needs. This ensures transparency and rigor in examining youth athletes’ experiences.

## 2. Materials and Methods

The current systematic review followed the Preferred Reporting Items for Systematic Reviews and Meta-Analyses guidelines [41]. The study protocol was registered with Open Science Framework (OSF) at the following link: https://doi.org/10.17605/OSF.IO/EGKWM (accessed on 3 December 2024).

### 2.1. Eligibility Criteria

This review adhered to the population, interventions, comparisons, outcomes, and study design (PICOS) framework [42] to identify the essential concepts of the studies related to the research question in advance and to streamline the search process. The following inclusion criteria were applied: (i) empirical articles written in English; (ii) studies conducted with young male football players aged between 13 and 18 years (adolescents only) or 13 y ≥ Mage ≤ 18 y, when the age range was not clear. Studies including participants outside this age range were excluded unless the overall sample met the specified mean age criterion; (iii) articles that contain relevant information about the variables being analyzed; (d) published after 1985. The following exclusion criteria were employed: (i) studies that included participants from sports other than football; (ii) studies that involved female athletes; (iii) Studies that lacked relevant data on the psychological aspects under investigation; (iv) abstracts, conference proceedings, book chapters, systematic reviews, and meta-analyses were also excluded, as they did not align with the focus of this sample.

### 2.2. Sources of Information, Research

For this systematic review, the search was carried out on 4 November 2024, on the following electronic databases: Web of Science (all databases), Scopus, ERIC, and SportDiscus. The search strategy applied was: ‘(Football* OR Soccer) AND (you* OR talent* OR adolescent* OR child*) AND (passion OR “autonomous motivation” OR motiv* OR “self-determin*” OR “life satisfaction” OR “assessment of satisfaction” OR “satisfaction with life” OR “positive affect” OR “negative affect” OR enjoy* OR “basic psychological needs” OR “behavior regulation”).

Table 1 outlines the search strategy used for each database. Additionally, the reference lists of the selected studies were examined to identify any relevant articles that may not have been captured in the initial search. When full-text articles were not accessible in the databases, the authors were contacted directly to request the complete versions. Alongside the database searches, a manual prospective citation search was also conducted. The articles were imported into EndNote X9.3.3 software (Clarivate Analytics, Philadelphia, PA, USA), where gray literature was excluded, and duplicates were automatically and manually removed.

### 2.3. Data Extraction

Data extraction was carried out by two authors (DB and CM) using a standardized approach (see Table 5) about the studies’ characteristics (authors’ names and year of study publication, study aim, participants, the instruments used, the statistical analyses performed, the results obtained, and the main conclusions drawn). A third author (HS) was responsible for resolving potential discrepancies.

### 2.4. Data Items, Synthesis, and Charting

Once the final selection of articles was made, data from eligible articles were extracted and organized in a Microsoft Excel file. The data categories included: author(s), year of publication, article title, type of study (e.g., cross-sectional, cohort, longitudinal), Study Goals, sample (size and average age), Instruments, Statistical Analysis, results, and main conclusions. In this process, the articles were grouped according to the Type of Study.

### 2.5. Risk of Bias and Study Quality Assessment

The methodological quality of longitudinal and cohort studies was evaluated using the Newcastle–Ottawa Quality Assessment Form (NOS) [43,44,45], which uses a star-based scoring system across three domains: Selection, Comparability, and Outcome. The Selection domain assesses participant recruitment and sample representation, Comparability evaluates control of confounders, and Outcome measures the consistency of outcomes and adequacy of follow-up. Studies received quality ratings of “good,” “fair,” or “poor” based on their star scores in these domains. Cross-sectional studies were assessed using the Joanna Briggs Institute (JBI) Critical Appraisal Tools [46,47,48], which include an eight-item checklist aligned with the GRADE framework. Each item is scored as “Yes” (1), “No” (0), “Unclear” (0), or “Not applicable” (not scored). Studies scoring below 50% (4 points) were excluded. For Vallerand’s [49] narrative study, the SANRA scale [50] will be used, which consists of six items rated from 0 (low quality) to 2 (high quality), with a maximum score of 12. This scale evaluates the review’s significance, objectives, literature search description, reference accuracy, scientific reasoning, and presentation of relevant data.

## 3. Results

### 3.1. Study Selection/Description of Studies

**Figure 1:**
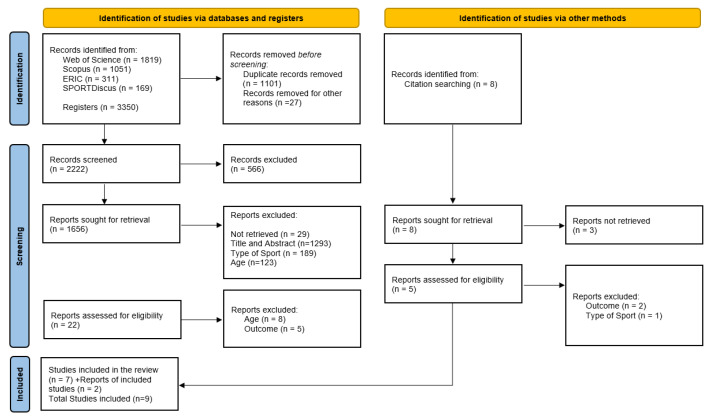
PRISMA diagram for the identification, screening, eligibility, and inclusion of studies.

The literature search was conducted across the Web of Science, Scopus, ERIC and SPORTDiscus databases, producing 3350 articles. Following the PRISMA guidelines, the search and selection process was conducted independently by D.B. and C.M. The initial database search using the keywords passion, motivation, affect, and satisfaction identified 3350 results. Duplicate articles were removed using EndNote X9.3.3 software (Clarivate Analytics, Philadelphia, PA, USA). After analyzing the titles, 1656 potentially relevant studies were identified (Figure 1). Following a review of the abstracts, 22 articles were selected based on pre-defined inclusion and exclusion criteria. Upon reading the full texts, 7 studies were deemed suitable for inclusion in the final sample. Additionally, through citation searching, 2 more articles were identified. In total, 9 articles were included, of which 7 (77.8%) were cross-sectional and 2 (22.2%) were longitudinal. An analysis of the quality of the studies (Table 2 and Table 3) revealed that six studies were of good quality [51,52,53,54,55,56], one study was of reasonable quality [57], and both longitudinal studies [34,49] were included. All the selected studies (Table 1) articles were published between 2011 and 2022. Out of a total of nine articles, two are from the UK [55,56], three from Spain [52,53,54], one from the United States [34], one from Scotland [57], one from Norway [51], and one from Canada [49] (Table 4).

Among the nine studies analyzed, only one did not examine the variable of passion [57]. Five studies focused on the satisfaction of basic needs [34,52,53,54,55]. Collectively, the articles involved a total of 2062 individuals aged between 11 and 18 years. The studies primarily explored the relationships between passion (both harmonious and obsessive), motivation, and athlete burnout. Some research investigated the mediating role of psychological need satisfaction in these relationships, while others examined how passion impacts performance and well-being. However, few studies addressed all constructs simultaneously.

### 3.2. Main Findings

This study examined the relationships among passion, motivation, psychological need satisfaction, burnout, affect, and performance in young football athletes. Harmonious passion (HP) has been shown to have a consistently protective and performance-enhancing effect, while obsessive passion (OP) has demonstrated a weaker or maladaptive association. Path analyses revealed a substantial impact of HP on self-determined motivation, with a significant coefficient of γ = 0.43, *p* < 0.01 [56]. In comparison, the coefficient of OP was negligible (γ = 0.04, *p* > 0.05) [56]. Self-determined motivation significantly predicted lower burnout (β = 0.64, *p* < 0.01), supporting an indirect pathway through which HP reduces burnout [56]. Bivariate correlations confirmed that HP was negatively related to all burnout dimensions, while OP was unrelated to exhaustion, and HP positively predicted psychological need satisfaction, which in turn negatively predicted burnout [55].

Autonomous motivation and the self-determination index (SDI) demonstrated the highest levels across the sample, although U17 athletes exhibited lower autonomous motivation compared to U15 and U13 athletes. Additionally, years in the academy for U17 athletes were associated with decreased self-determined motivation and increased controlled motivation [57]. The hours of football practice were largely unrelated to motivation. Mediation analyses demonstrated that satisfaction of autonomy, competence, and relatedness predicted both HP and OP via autonomous motivation, with relatedness satisfaction specifically reducing OP through controlled motivation [52]. Research indicates a strong correlation between fulfilling basic psychological needs and reducing burnout (r = −0.57 to −0.21). Additionally, the satisfaction of these needs indirectly impacts reflection and concentration via HP and OP, with direct effects of autonomy and competence on performance outcomes [53] (Table 5).

**Table 5:** Results.

Author	Type of Study	Study Goals	Sample	Instruments	Statistical Analysis	Results	**Main Conclusions**
Curran, T. et al., 2011 [56]	Cross-sectional Study	Examine how harmonious and obsessive passion relates to athlete exhaustion and if these relationships are mediated by self-determined motivation.	149 youths (M = 16.2, s = 2.0, range = 12–21)	Passion Scale; Sport Motivation Scale (SMS); Athlete Burnout Questionnaire (ABQ).	Confirmatory Factor Analysis; Path Analysis	Path Analysis Summary: The path coefficient between harmonious passion and self-determined motivation was significant (y = 0.43, *p* < 0.01), while obsessive passion’s coefficient was non-significant (y = 0.04, *p* > 0.05). Self-determined motivation significantly predicted burnout (β = 0.64, *p* < 0.01). Mediation Models: -Model 1 (M1): Only direct paths were analyzed; results showed a non-significant relationship between obsessive passion and burnout (y = −0.07, *p* > 0.05) and a significant negative relationship between harmonious passion and burnout (y = −0.26, *p* < 0.01). -Model 2 (M2): All paths except for obsessive passion were significant, with no direct paths from passion to burnout. -Model 3 (M3): Included direct paths. The fit of M2 and M3 did not differ significantly (Δ1.23 (2), *p* > 0.05), and the direct effect of harmonious passion on burnout was non-significant (β = −0.01, *p* > 0.05).	Most participants scored low to moderate on burnout subscales. Obsessive passion showed no relation to self-determination and athlete burnout symptoms. When controlling for obsessive passion, harmonious passion was positively linked to self-determination and negatively associated with reduced accomplishment. Harmonious passion inversely predicted athlete burnout through self-determined motivation, while no mediation was found for obsessive passion.
Curran, T. et al., 2013 [55]	Cross-sectional Study	Examine the mediating role of psychological need satisfaction in relationships between types of passion for sport and athlete burnout	173 young male soccer players (M = 15.46, s = 1.47, range = 13–18 years)	Vallerand et al.’s passion scale; Autonomy (Standage, Duda, and Ntoumanis); Competence (subscale of the Intrinsic Motivation Inventory); Relatedness (Richer and Vallerand’s acceptance scale). Athlete Burnout Questionnaire (ABQ);	Descriptive and Inferential Quantitative Analysis; Path Analysis	Bivariate correlations revealed that harmonious passion had a negative relationship with all burnout dimensions and total burnout (r = −0.21 to r = −0.09, *p* < 0.05). Obsessive passion showed no relation to exhaustion (r = −0.00). Composite psychological need satisfaction was inversely associated with reduced accomplishment (r = −0.57), exhaustion (r = −0.21), devaluation (r = −0.31), and total burnout (r = −0.42, all *p* < 0.01). The path coefficient for harmonious passion and psychological need satisfaction was significant (y = 0.20, *p* < 0.01), while obsessive passion was non-significant (y = 0.04, *p* > 0.05). Psychological need satisfaction negatively influenced burnout dimensions, with significant path coefficients for reduced accomplishment (β = −0.53), exhaustion (β = −0.21), and devaluation (β = −0.31), as well as total burnout (β = −0.42, all *p* < 0.01).	Psychological need satisfaction mediated the relationship between harmonious passion and athlete burnout but not obsessive passion and athlete burnout; The Inverse relationship between harmonious passion and burnout can be explained by higher levels of psychological need satisfaction, contrary to obsessive passion.
Hendry, D. et al., 2014 [57]	Quantitative, Cross-sectional study with elements of observational and survey-based research	Explore the link between childhood play, practice hours, and motivation in elite youth soccer players, focusing on how self-led play and coach-led practice influence motivation.	Male, elite youth soccer players (N = 144), 11–16 years; U13 (12.0–12.9 year, *n* = 46), U15 (14.0–14.9 year, *n* = 50) and U17 (15.0–16.9 year, *n* = 48)	BRSQ (Behavioral Regulation in Sport Questionnaire); Specific soccer practice questionnaire.	One-way ANOVA; Mixed ANOVA with repeated measures; Post hoc Tukey HSD tests; Greenhouse-Geisser corrections; Partial eta-squared	SDI and autonomous motivation exhibited the highest motivation indices (SDI—16.37 ± 3.93; AM—24.74 ± 2.06). Group U17 showed lower autonomous motivation (M = 24.74, SD = 2.08) compared to U15 (M = 26.02, SD = 2.08) and U13 (M = 26.42, SD = 1.79). No significant correlations were found between accumulated hours of playing and practicing football and motivation indices, except for a relationship between practice hours and integrated regulation (r = 0.18). Positive correlations were observed between hours in play and training for U15 (r = 0.38, *p* < 0.01) and U17 (r = 0.31, *p* < 0.05). For U17, years at the academy negatively impacted SDI (R2change = 0.19, b = −0.72, *p* < 0.05) while positively affecting controlled extrinsic motivation (R2change = 0.13, b = 0.58, *p* < 0.05).	It was not possible to verify that the hours spent playing during childhood were positively related to the current levels of elite athletes. There are no associations between current levels of intrinsic or autonomous motivation and play.
Chamorro, J. L. et al., 2016 [54]	Cross-sectional Quantitative Study	Examine variations in future achievements (sports, academics, personal life) and differences in passion, motivation, and psychological needs among young elite soccer players.	478 young male elite soccer players (Mage 17.42, SD = 0.705, Range = 15–19)	A questionnaire with a scale adapted from the Dual Career Survey (DCS); Spanish version of the Passion Scale; Spanish adaption of the Behavioral Regulation in Sport Questionnaire (BRSQ); Spanish adaptations of the autonomy satisfaction scale;	Descriptive Statistics; Pearson Correlation Analysis; Cluster Analysis; MANOVA	The life spheres balance group shows higher levels of harmonious passion (η^2^ = 0.06, F(2, 475) = 9.990, *p* < 0.001) than the players of the other groups; The life spheres balance group shows higher levels of autonomous motivation (η^2^ = 0.10, F(2, 475) = 13.597, *p* < 0.001), autonomy (η^2^ = 0.07, F(2, 475) = 6.592, *p* < 0.01) and relatedness satisfaction (η^2^ = 0.07, F(2, 475) = 5.603, *p* < 0.01) than the sport-oriented group as well as lower levels of amotivation (η^2^ = 0.04, F(2, 475) = 6.665, *p* < 0.01) than the private life oriented group.	A profile emphasizing future achievements in sports, academics, and personal life is linked to higher passion, motivation, and satisfaction of basic psychological needs. Among the Sport-Oriented and Life Spheres Balance groups, becoming a professional in their passion was the top future goal, with notable differences in passion, motivation, and basic psychological needs satisfaction observed between the two groups.
Verner-Filion, J. & Vallerand, R., 2018 [34]	Longitudinal Study	Investigate how athletes’ optimal functioning (positive/negative affect, satisfaction, and performance quality) varies with passion types and need satisfaction over three competitive seasons.	116 male youth soccer players, ranging from 12 to 24 years of age (M = 16.02 years, SD = 2.59)	Passion Scale; Basic Need Satisfaction Scale; Positive and Negative Affect Scale; Satisfaction With Life Scale; Coach’s evaluation (scale from 0 to 10); Coach’s evaluation (27-item questionnaire, 5-point Likert scale).	Hierarchical Linear Modeling—HLM	Harmonious Passion (HP) was positively linked to positive affect (γ01 = 0.12, *p* = 0.005), while Obsessive Passion (OP) showed no relation. Within-person satisfaction of autonomy, relatedness, and competence was positively correlated with positive affect (γ10 = 0.20, *p* < 0.001; γ20 = 0.15, *p* = 0.004; γ30 = 0.18, *p* < 0.001). HP negatively associated with negative affect (γ01 = −0.23, *p* = 0.017), whereas OP was positively related (γ02 = 0.13, *p* = 0.022). Autonomy satisfaction was also negatively tied to negative affect (γ10 = −0.15, *p* = 0.034). OP, but not HP, negatively impacted athletic satisfaction changes (γ02 = −0.15, *p* < 0.001), while satisfaction in these areas positively affected athletic satisfaction (γ10 = 0.32, *p* < 0.001; γ20 = 0.32, *p* < 0.001; γ30 = 0.13, *p* = 0.031). HP uniquely predicted preparation quality (γ01 = 0.42, *p* = 0.021), with competence satisfaction also positively correlating (γ30 = 0.46, *p* = 0.004).	The more athletes were harmoniously passionate toward soccer, the higher levels of positive affect they reported; athletes who experienced higher satisfaction of all three basic psychological needs at each measurement point reported higher levels of positive affect on a within-person basis; athletes who experienced higher satisfaction of all three basic psychological needs at each measurement time reported higher levels in athletic satisfaction
Chamorro, J. L. et al., 2020 [53]	Cross-sectional Quantitative Study	Investigate how athletes’ optimal functioning (positive/negative affect, satisfaction, and performance quality) varies with passion types and needs satisfaction over three competitive seasons.	478 young male elite football players (Mage 17.42, SD = 0.705, range = 16–19)	Autonomy Satisfaction Scale; Intrinsic Motivation Inventory (IMI); Perceived relatedness scale; Passion Scale. Reflective Learning Continuum (RLC); Subscale of Concentration Disruption from the Sport Anxiety Scale-2	WLSMV Estimator with MPLUS 7.0; Confirmatory Factor Analysis; Exploratory Structural Equation Modeling (ESEM); Model fit assessed using χ^2^/gl, RMSEA, CFI, TLI, and SRMR.	Except for OP-autonomy (r = 0.02), OP-relatedness satisfaction (r = 0.01), and autonomy satisfaction-concentration disruption (r = −0.01), all correlations were significant. Both Model Partial Mediation (MPM) and Model Complete Mediation (MCM) fit the data well. Key findings include: -Autonomy satisfaction was positively related to reflection (A → HP → RE = 0.06) and negatively to concentration disruption (A → HP → CD = −0.05), with a direct positive effect on concentration disruption (A → CD = 0.25). -Competence satisfaction had a positive indirect effect on reflection (C → HP → RE = 0.16) and a negative effect on concentration disruption (C → HP → CD = −0.18), along with a positive indirect effect on concentration disruption through OP (C → OP → CD = 0.09) and a direct effect on reflection (C → RE = 0.08). -Relatedness satisfaction showed a positive indirect effect on reflection (R → HP → RE = 0.22) and a negative effect on concentration disruption (R → HP → CD = −0.16), with a direct effect on reflection (R → RE = 0.11) and a negative direct effect on concentration disruption (R → CD = −0.24).	Obsessive passion mediates the positive relationship between competence, satisfaction and concentration disruption. Competence and relatedness satisfaction directly influence reflection positively, while relatedness satisfaction negatively impacts concentration disruption. Only harmonious passion mediates the influence of young footballers’ environment on their performance through psychological needs satisfaction. Perceived competence affects concentration disturbance positively when mediated by obsessive passion, with relational satisfaction distinguishing between reflection and concentration disruption.
Chamorro, J. L. et al., 2021 [52]	Cross-sectional observational study	Investigate how players’ satisfaction of three basic psychological needs predicts harmonious and obsessive passions through autonomous and controlled motivations.	478 young male football players (Mage = 17.43, SD = 0.71; range = 15‒19)	Spanish versions of scales assessing autonomy, competence, and relatedness; Spanish version of the Behavioral Regulation in Sport Questionnaire (BRSQ); Spanish adaptation of the Passion Scale	Structural Equation Modeling—SEM	Autonomous motivation had positive correlations with autonomy satisfaction (r = 0.57) and relatedness satisfaction (r = 0.47), and a high correlation with Harmonious Passion (HP) (r = 0.66). HP also correlated positively with basic needs: autonomy (r = 0.41), competence (r = 0.44), and relatedness (r = 0.54), as well as with Obsessive Passion (OP) (r = 0.44). Autonomy and relatedness satisfaction were moderately correlated (r = 0.50). Model Partial Mediation (MPM) and Model Complete Mediation (MCM) showed an excellent fit. MPM indicated that autonomy satisfaction was indirectly related to HP (A → AR → HP [95% CI] = 0.20 [0.12, 0.29]) and OP (A → AR → OP [95% CI] = 0.09 [0.02, 0.16]) through autonomous motivation. Competence satisfaction had positive indirect effects on HP (C → AR → HP [95% CI] = 0.09 [0.04, 0.14]) and OP (C → AR → OP [95% CI] = 0.04 [0.00, 0.08]), and direct effects on both HP (C → HP = 0.20) and OP (C → OP = 0.17). Relatedness satisfaction showed positive indirect effects on HP (R → AR → HP [95% CI] = 0.09 [0.02, 0.16]) and OP (R → AR → OP [95% CI] = 0.04 [0.00, 0.08]), a negative indirect effect on OP through controlled motivation (R → CR → OP [95% CI] = −0.11 [−0.17, −0.05]), and a positive direct effect on HP (R → HP = 0.25).	Autonomous and controlled regulations mediate the link between psychological needs and passion. Satisfaction of autonomy and competence directly and indirectly affects two passions: obsessive passion (OP) and harmonious passion (HP). Autonomous regulations positively predict both OP and HP, with a stronger effect on HP. Furthermore, meeting players’ basic needs foster harmonious passion and has mixed effects on obsessive passion, illustrated through direct and mediated relationships involving behavioral regulations.
Sigmundsson, H. et al., 2022 [51]	Cross-sectional Observational	Examine differences in passion, grit, and mindset between elite and junior football teams.	46 male athletes Elite: mean age 22.32 (SD = 4.86, N = 25); Junior: mean age 14.85 (SD = 0.35, N = 21)	Passion scale; Norwegian version of the Grit S Scale; Norwegian version of Theories of Intelligence Scale (TIS).	Comparative analysis between groups using the Mann–Whitney U test	**Elite group:** Passion total score HFC (M = 4.75, SD = 0.22) > LFC (M = 4.42, SD = 0.52) (*p* = 0.04). There was no significant difference between the groups about grit (M = 4.17, SD = 0.22 vs. M = 3.98, SD = 0.46) or mindset (M = 4.40, SD = 0.45 vs. M = 4.32, SD = 1.09). **Junior group:** Passion total score HFC (M = 4.87, SD = 0.20) > LFC (M = 4.46, SD = 0.35) (*p* = 0.004). There was no significant difference between the groups about grit (M = 3.98, SD = 0.65 vs. M = 3.64, SD = 0.39) or mindset (M = 4.69, SD = 1.11 vs. M = 4.70, SD = 0.75)	Passion distinguishes elite players by enhancing competence and performance. While grit may not significantly separate the best from the good, it can help achieve high performance. Mindset influences results, but there is no difference in mindset between high and low-performance groups.
Vallerand, R., 2012 [49]	Longitudinal Study; Descriptive	Understand how motivation and passion contribute to a meaningful life; Examine the influence of social factors on intrinsic motivation; Integrate personality, task, and social factors in motivational processes; Explore the role of passion through the dualistic model (harmonious vs. obsessive).	The article does not specify a singular sample for a study but instead summarizes a series of research initiatives conducted by Robert J. Vallerand and his team over a span of approximately 30 years.	Academic Motivation Scale; Leisure Motivation Scale; Sport Motivation Scale; Self-Determination Theory Measures; Passion Scale	Path Analysis; Exploratory and Confirmatory Factor Analyses; Structural Equation Modeling (SEM)	Research indicated that individuals’ perceptions of how others (like parents and teachers) behave towards them significantly affect their motivation and subsequent outcomes. The actual social environment’s influence can trigger motivational sequences that lead to meaningful outcomes; The study found that harmonious passion positively predicts psychological well-being, life satisfaction, and vitality, while obsessive passion correlates with negative outcomes like anxiety and depression; A high prevalence of passion was observed, with about 84% of participants reporting a passion for at least one activity, engaging an average of 8.5 h per week; The research successfully developed a valid Passion Scale consisting of two subscales (harmonious and obsessive passion) demonstrating strong reliability and construct validity across various studies; Harmonious passion contributes positively to psychological well-being and physical health, while obsessive passion may lead to negative psychological outcomes, emphasizing the importance of the quality of motivational processes; The findings underscore that motivation is influenced by various factors, including personal characteristics, social environment, and the context of activities. The quality of motivation (e.g., intrinsic versus extrinsic) matters significantly for outcomes.	Motivational processes are central to living a meaningful life. Research shows that self-determined motivation and harmonious passion foster positive outcomes, while extrinsic motivation and obsessive passion often relate to negative ones. The dualistic model of passion offers a useful framework for understanding these differences. Social environments, particularly the influence of parents, teachers, and peers, play a key role in shaping motivation and well-being. The validated Passion Scale provides a reliable tool to distinguish between harmonious and obsessive passion. Future studies should further explore how different motivational processes and contexts interact to impact life outcomes.

Athletes who maintain balanced engagement across life domains have been shown to have higher HP, autonomous motivation, and satisfaction of autonomy and relatedness, alongside lower amotivation. This suggests that maintaining a balanced lifestyle enhances motivation and well-being [54]. HP also predicted positive effects on mood and athlete satisfaction, while OP was linked to negative effects. Satisfaction with autonomy, competence, and relatedness had a further influence on affective states and perceived preparation quality [34,52]. HP uniquely predicted preparation quality and sport engagement, with high-commitment athletes scoring higher in passion than low-commitment athletes, and no differences in grit or mindset [34,51]. Extant literature consistently associates HP with well-being, vitality, and adaptive performance, and OP with anxiety, depression, and maladaptive outcomes. This underscores the importance of motivation quality for positive psychological and performance results [49]. Collectively, the findings suggest a pathway from HP to psychological need satisfaction, to autonomous motivation, ultimately reducing burnout, enhancing affective well-being, and improving performance. In contrast, OP has weaker or negative effects. Factors such as age and context, including reduced autonomous motivation in older athletes and the benefits of a balanced lifestyle, further moderate these relationships.

## 4. Discussion

This study aimed to provide a comprehensive understanding of the relationship between passion, motivation, well-being, and performance among young athletes. Building on the Dualistic Model of Passion and Self-Determination Theory, the findings from the current literature converge toward a consistent conclusion: harmonious passion (HP) and self-determined motivation operate as powerful protective and performance-enhancing factors, whereas obsessive passion (OP) and controlled motivation appear linked to maladaptive outcomes.

Empirical evidence demonstrates that HP fosters greater psychological well-being [12,58,59], satisfaction of basic psychological needs (BPN) [58,59,60], and more adaptive emotional experiences, allowing athletes to maintain high levels of engagement without compromising mental health [58,60,61]. Athletes driven by HP experience higher resilience, lower risk of burnout, and greater flexibility in their investment in sport, as passion stems from intrinsic enjoyment and volitional engagement rather than internal or external pressure [62,63,64]. Conversely, OP has been systematically associated with negative emotions, need frustration, diminished well-being, and increased burnout risk, particularly when participation becomes compulsive or tied to external validation [62,65].

The protective function of HP becomes evident at high pressure. HP enables athletes to adopt a “challenge mindset,” viewing high-pressure situations as opportunities for growth rather than threats, which helps buffer against negative emotions and supports adaptive functioning during competitions [62]. In football, higher HP is associated with better performance, greater well-being, and more deliberate practice, all of which contribute to success in high-pressure situations [51]. These protective effects are consistently linked to lower levels of athlete burnout and exhaustion [66,67], supporting adaptive coping [56,68] and resource recovery [69]. These mechanisms highlight that HP is not only linked to emotional well-being but also to sustainable motivation, enabling individuals to remain committed and persistent without sacrificing mental health.

In performance terms, HP and self-determined motivation predict higher achievement, vigor, confidence, and sustained effort [70,71,72]. Research shows that the highest levels of sport engagement and performance are found among athletes with high HP, rather than those simply high in overall passion [73]. HP promotes flow experiences, enjoyment, strong athletic identity, and consistent training habits, creating a virtuous cycle that strengthens both performance and well-being [74,75,76]. Importantly, dedication and commitment have been identified as mediators in this process, strengthening the association between HP and athlete identity [77,78].

This pattern aligns with Self-Determination Theory, whereby satisfaction of autonomy, competence, and relatedness nurtures self-determined motivation and HP, facilitating increased resilience, vitality, and well-being [79,80]. Correspondingly, controlled motivation and amotivation, often accompanying O, predict exhaustion, negative affect, and withdrawal from sport, reinforcing the importance of psychological need satisfaction in preventing burnout [81,82,83]. The motivational environment emerges as a crucial determinant of these outcomes. Task-involving (mastery) climates and autonomy-supportive coaching styles foster need satisfaction, HP, and motivation sustainability, whereas ego-involving climates and controlling leadership predict burnout and motivational decline [79,84,85,86]. Such findings are particularly relevant for older youth athletes, among whom declines in intrinsic motivation are not a fixed developmental pattern, but rather a consequence of competitive pressure, inequalities in progression, and lack of psychological support [87,88,89].

Beyond passion and motivation alone, regulatory focus also plays a crucial role in enhancing athlete safety and performance. A prevention focus (PH) supports injury-preventive behaviors and risk-management practices [90,91,92]. Meanwhile, the effects of promotion focus (PO) depend heavily on regulatory fit: promotion-focused strategies enhance motivation only when intervention and athlete orientation are aligned; otherwise, they may impair performance or safety [93,94]. These results underscore the importance of individualized motivational approaches over universal prescriptions. Recent studies further emphasize that motivational profiles, rather than absolute practice volume, predict mental health, anxiety, self-esteem, persistence, and dropout risk [77,87,95]. Tailored interventions based on motivational profiles are associated with improved performance, engagement, and psychological outcomes [96].

### 4.1. Practical Implications

The findings emphasize key considerations for designing youth sports environments. Firstly, interventions should avoid attempts to reduce commitment or passion, as these factors do not predict burnout. Coaches, clubs, and parents ought to create autonomy-supportive environments that encourage voluntary participation and recognize personal development goals. Involving young athletes in decision-making regarding training, roles, or recovery can enhance their intrinsic motivation.

Secondly, programs that provide constructive feedback and cultivate mastery-oriented climates can improve both performance and overall satisfaction of needs, particularly for athletes under 17, who often experience a decline in motivation. Coaching should prioritize learning and personal growth instead of solely focusing on performance rewards, which can lead to a shift toward controlled motivation. Finally, promoting a balanced lifestyle, encompassing school, friendships, and leisure activities, offers psychological protection and results in higher performance and well-being. Clubs and federations should establish guidelines for adequate rest, academic support, and extracurricular opportunities to foster this balance.

### 4.2. Study Limitations

Methodological limitations must be acknowledged. The limited number of studies (*n* = 9) restricts the generalizability of the findings, as the comprehensive inclusion and exclusion criteria narrowed the eligible studies. This rigorous selection did allow for a focused analysis of a specific population of youth football players. However, the predominance of cross-sectional designs limits broad conclusions. The lack of longitudinal studies hinders the ability to examine developmental trajectories and long-term effects of passion, motivation, and psychological need satisfaction. Additionally, the diversity in research designs prevented quantitative meta-analysis, limiting statistical synthesis of effect sizes. Most subjects were male, restricting conclusions about gender differences in passion and burnout. Contextual factors like socioeconomic status and parental involvement were often unmeasured, despite their potential influence on motivation and athlete development. Furthermore, the focus on individual cultural contexts limits generalizability globally due to variability in youth sport participation across countries. These methodological concerns highlight the value of adopting a broader approach in future reviews, including more diverse inclusion criteria, a higher prevalence of longitudinal studies, and a heterogeneous sample. This could enhance the generalizability and reliability of findings related to passion, motivation, and well-being in youth football.

### 4.3. Future Research

Future research should focus on longitudinal designs to track changes in passion and need satisfaction during key developmental phases. It is essential to investigate causal mechanisms through randomized autonomy-support interventions and specialized coach training. With a concerning decline in autonomous motivation in academia, we must determine if this arises from environmental pressures, trait changes, or identity conflicts. Additionally, gender-comparative studies are important since girls face unique motivational and s challenges in competitive sports. Cross-cultural research should explore whether the benefits of high passion and need satisfaction are consistent across different football systems. Finally, examining cognitive and neurobiological factors—like stress reactivity and sleep patterns—is crucial for understanding the link between high-stakes sports and mental well-being.

## 5. Conclusions

This synthesis highlights that the sustainability of youth football participation relies more on motivational factors than on training intensity or commitment. Harmonious passion, autonomous motivation, and basic need satisfaction enhance well-being, positive emotions, and athletic satisfaction while protecting against burnout. In contrast, obsessive passion lacks psychological resilience and can lead to negative emotions and lower satisfaction. These findings stress the importance of creating autonomy-supportive and psychologically safe environments, allowing young players to remain passionate while maintaining a healthy identity balance. By prioritizing motivational quality over mere intensity, coaches and organizations can promote both high performance and long-term mental well-being in youth football.

## Figures and Tables

**Table 1:** Sources of Information, Research.

Database	Syntax	Number
Web of Science (all databases)	Football* OR Soccer (Topic) AND you* OR talent* OR adolescent* OR child* (Topic) AND passion OR “autonomous motivation” OR motiv* OR “self-determin*” OR “life satisfaction” OR “assessment of satisfaction” OR “satisfaction with life” OR “positive affect” OR “negative affect” OR enjoy* OR “basic psychological needs” OR “behavior regulation” (Topic)	1819
Scopus	(TITLE-ABS-KEY (football* OR soccer) AND TITLE-ABS-KEY (you* OR talent* OR adolescent* OR child*) AND TITLE-ABS-KEY (passion OR “autonomous motivation” OR motiv* OR “self-determin*” OR “life satisfaction” OR “assessment of satisfaction” OR “satisfaction with life” OR “positive affect” OR “negative affect” OR enjoy* OR “basic psychological needs” OR “behavior regulation”)	1051
ERIC	(Football* OR Soccer) AND (you* OR talent* OR adolescent* OR child*) AND (passion OR “autonomous motivation” OR motiv* OR “self-determin*” OR “life satisfaction” OR “assessment of satisfaction” OR “satisfaction with life” OR “positive affect” OR “negative affect” OR enjoy* OR “basic psychological needs” OR “behavior regulation”)	311
SPORTDiscus	TI (Football* OR Soccer) AND AB (you* OR talent* OR adolescent* OR child*) AND AB (passion OR “autonomous motivation” OR motiv* OR “self-determin*” OR “life satisfaction” OR “assessment of satisfaction” OR “satisfaction with life” OR “positive affect” OR “negative affect” OR enjoy* OR “basic psychological needs” OR “behavior regulation”)	169

**Table 2:** Quality analysis of cross-sectional studies.

	JBI Critical Appraisal Checklist for Analytical Cross-Sectional Studies									
	1.	2.	3.	4.	5.	6.	7.	8.	Total	Overall Appraisal
Curran, T. et al., 2011 [56]	Unc	Yes	Yes	Yes	Unc	No	Yes	Yes	5	I
Curran, T. et al., 2013 [55]	Unc	Yes	Yes	Yes	No	No	Yes	Yes	5	I
Hendry, D. et al., 2014 [57]	Yes	Yes	Unc	Yes	Unc	Yes	Yes	Yes	6	I
Chamorro, J.L. et al., 2016 [54]	Yes	Yes	Yes	Yes	Unc	No	Yes	Yes	6	I
Chamorro, J.L. et al., 2020 [53]	Yes	Yes	Yes	Yes	Unc	No	Yes	Yes	6	I
Chamorro, J.L. et al., 2021 [52]	Yes	Yes	Yes	Yes	Unc	No	Yes	Yes	6	I
Sigmundsson, H. et al., 2022 [51]	Unc	No	Yes	Yes	Unc	Yes	Yes	Yes	5	I

Legend: Yes; No; Unc—Unclear; I—Include.

**Table 3:** Quality analysis of longitudinal and cohort studies.

	Newcastle-Ottawa Quality Assessment Form (NOS)								
	Selection				Comparability	Outcome			
	1.	2.	3.	4.	5	6	7	8	Quality
Verner-Filion, J. & Vallerand, R., 2018 [34]		X		X	X	X	X	X	Fair

Legend: Good quality: 3 or 4 stars in the selection domain AND 1 or 2 stars in the comparability domain AND 2 or 3 stars in the outcome/exposure domain; Fair quality: 2 stars in the selection domain AND 1 or 2 stars in the comparability domain AND 2 or 3 stars in outcome/exposure domain; Poor quality: 1 star in selection domain OR 0 stars in comparability domain OR 0 or 1 stars in outcome/exposure domain.

**Table 4:** Quality analysis of narrative review studies-SANRA scale.

	SANRA Scale						
	1.	2.	3.	4.	5	6	Quality
Vallerand, R., 2012 [49]	2	2		2	2	1	9/12

## Data Availability

Data are contained within the article.

## References

[1] Murthy V.H. (2023). Physical Activity: An Untapped Resource to Address Our Nation’s Mental Health Crisis Among Children and Adolescents. Public Health Rep..

[2] Verner-Filion J., Gaudreau P. (2023). Need satisfaction and sport motivation predict selection in a competitive youth soccer team over and above the relative age effect. Int. J. Sport Psychol..

[3] Vancakova J., Chamarro A., Martínez-Martí M.L. (2021). Does Passion mediate the Effect of Character Strengths on the Resilience of Athletes?. Cuad. Psicol. Deporte.

[4] Rodrigues F., Monteiro D., Matos R., Jacinto M., Antunes R., Amaro N. (2023). Exploring the Dynamics of Athletes’ Enjoyment and Self-Determined Motivation, and of the Motivational Climate in Youth Football: A Longitudinal Perspective. Percept. Mot. Ski..

[5] Vallerand R.J., Blanchard C., Mageau G.A., Koestner R., Ratelle C., Leonard M., Gagne M., Marsolais J. (2003). Les passions de l’ame: On obsessive and harmonious passion. J. Pers. Soc. Psychol..

[6] Curran T., Hill A.P., Hall H.K., Jowett G.E. (2015). Relationships Between the Coach-Created Motivational Climate and Athlete Engagement in Youth Sport. J. Sport Exerc. Psychol..

[7] Vallerand R.J. (2008). On the Psychology of Passion: In Search of What Makes People’s Lives Most Worth Living. Can. Psychol..

[8] Stenseng F., Dalskau L.H. (2010). Passion, Self-Esteem, and the Role of Comparative Performance Evaluation. J. Sport Exerc. Psychol..

[9] Curran T., Hill A.P., Appleton P.R., Vallerand R.J., Standage M. (2015). The psychology of passion: A meta-analytical review of a decade of research on intrapersonal outcomes. Motiv. Emot..

[10] Gustafsson H., Hill A.P., Stenling A., Wagnsson S. (2016). Profiles of perfectionism, parental climate, and burnout among competitive junior athletes. Scand. J. Med. Sci. Sports.

[11] Rodrigues F., Mageau G.A., Lemelin E., Teixeira D., Vitorino A., Cid L., Monteiro D. (2022). Life satisfaction of Paralympians: The role of needs satisfaction and passion. Int. J. Sports Sci. Coach..

[12] Bento T., Vitorino A., Cid L., Monteiro D., Couto N. (2024). Analysing the Relation between Passion, Motivation, and Subjective Well-Being in Sport: A Systematic Review. Sports.

[13] Lafrenière M.-A., St-Louis A., Vallerand R., Donahue E. (2012). On the Relation between Performance and Life Satisfaction: The Moderating Role of Passion. Self Identity.

[14] St-Louis A.C., Carbonneau N., Vallerand R.J. (2016). Passion for a Cause: How It Affects Health and Subjective Well-Being. J. Personal..

[15] Ryan R.M., Deci E.L. (2017). Self-Determination Theory: Basic Psychological Needs in Motivation, Development, and Wellness.

[16] Deci E.L., Ryan R.M. (2000). The “What” and “Why” of Goal Pursuits: Human Needs and the Self-Determination of Behavior. Psychol. Inq..

[17] Edward L., Deci R.M.R. (1985). Intrinsic Motivation and Self-Determination in Human Behavior.

[18] Pelletier L.G., Fortier M.S., Vallerand R.J., Brière N.M. (2001). Associations Among Perceived Autonomy Support, Forms of Self-Regulation, and Persistence: A Prospective Study. Motiv. Emot..

[19] Monteiro D., Teixeira D.S., Travassos B., Duarte-Mendes P., Moutão J., Machado S., Cid L. (2018). Perceived Effort in Football Athletes: The Role of Achievement Goal Theory and Self-Determination Theory. Front. Psychol..

[20] Hodge K., Lonsdale C., Jackson S.A. (2009). Athlete Engagement in Elite Sport: An Exploratory Investigation of Antecedents and Consequences. Sport Psychol..

[21] Teixeira D.S., Rodrigues F., Vitorino A., Cid L., Bento T., Evmenenko A., Macedo R., Morales-Sánchez V., Monteiro D. (2023). The dualistic model of passion in adapted sport: A double-serial mediation analysis on satisfaction with life. Curr. Psychol..

[22] Monteiro D., Pelletier L., Moutão J.M., Teixeira D., Cid L. Examining the motivational determinants of enjoyment and the intention to continue of persistent competitive swimmers. Proceedings of the Self-Determination Theory 2019.

[23] Amorose A.J., Anderson-Butcher D. (2007). Autonomy-supportive coaching and self-determined motivation in high school and college athletes: A test of self-determination theory. Psychol. Sport Exerc..

[24] Sarrazin P., Vallerand R., Guillet E., Pelletier L., Cury F. (2002). Motivation and dropout in female handballers: A 21-month prospective study. Eur. J. Soc. Psychol..

[25] Ntoumanis N., Standage M. (2009). Motivation in physical education classes:A self-determination theory perspective. Theory Res. Educ..

[26] Quested E., Duda J. (2011). Perceived Autonomy Support, Motivation Regulations and the Self-Evaluative Tendencies of Student Dancers. J. Danc. Med. Sci. Off. Public Int. Assoc. Danc. Med. Sci..

[27] Álvarez M.S., Balaguer I., Castillo I., Duda J.L. (2009). Coach Autonomy Support and Quality of Sport Engagement in Young Soccer Players. Span. J. Psychol..

[28] Balaguer I., González L., Fabra P., Castillo I., Mercé J., Duda J.L. (2012). Coaches’ interpersonal style, basic psychological needs and the well- and ill-being of young soccer players: A longitudinal analysis. J. Sports Sci..

[29] Bartholomew K.J., Ntoumanis N., Ryan R.M., Bosch J.A., Thøgersen-Ntoumani C. (2011). Self-Determination Theory and Diminished Functioning:The Role of Interpersonal Control and Psychological Need Thwarting. Personal. Soc. Psychol. Bull..

[30] Rousseau F.L., Vallerand R.J. (2008). An Examination of the Relationship between Passion and Subjective Well-Being in Older Adults. Int. J. Aging Hum. Dev..

[31] Clancy R.B., Herring M.P., MacIntyre T.E., Campbell M.J. (2016). A review of competitive sport motivation research. Psychol. Sport Exerc..

[32] Mack D.E., Wilson P.M., Gunnell K.E., Gilchrist J.D., Kowalski K.C., Crocker P.R.E. (2012). Health-Enhancing Physical Activity: Associations with Markers of Well-Being. Appl. Psychol. Health Well-Being.

[33] Lemyre P.-N., Roberts G.C., Stray-Gundersen J. (2007). Motivation, overtraining, and burnout: Can self-determined motivation predict overtraining and burnout in elite athletes?. Eur. J. Sport Sci..

[34] Verner-Filion J., Vallerand R.J. (2018). A longitudinal examination of elite youth soccer players: The role of passion and basic need satisfaction in athletes’ optimal functioning. Psychol. Sport Exerc..

[35] Diener E. (2000). Subjective Well-Being: The Science of Happiness and a Proposal for a National Index. Am. Psychol..

[36] Diener E., Suh E., Lucas R., Smith H. (1999). Subjective Well-Being: Three Decades of Progress. Psychol. Bull..

[37] Sheldon K., Elliot A., Kim Y., Kasser T. (2001). What Is Satisfying About Satisfying Events? Testing 10 Candidate Psychological Needs. J. Personal. Soc. Psychol..

[38] Mageau G.A., Vallerand R.J. (2003). The coach–athlete relationship: A motivational model. J. Sports Sci..

[39] Hodge K., Henry G., Smith W. (2014). A Case Study of Excellence in Elite Sport: Motivational Climate in a World Champion Team. Sport Psychol..

[40] Jõesaar H., Hein V., Hagger M.S. (2012). Youth athletes’ perception of autonomy support from the coach, peer motivational climate and intrinsic motivation in sport setting: One-year effects. Psychol. Sport Exerc..

[41] Page M.J., McKenzie J.E., Bossuyt P.M., Boutron I., Hoffmann T.C., Mulrow C.D., Shamseer L., Tetzlaff J.M., Akl E.A., Brennan S.E. (2021). The PRISMA 2020 statement: An updated guideline for reporting systematic reviews. BMJ.

[42] Schardt C., Adams M.B., Owens T., Keitz S., Fontelo P. (2007). Utilization of the PICO framework to improve searching PubMed for clinical questions. BMC Med. Inform. Decis. Mak..

[43] Wells G.A., Shea B., O’Connell D., Peterson J., Welch V., Losos M., Tugwell P. The Newcastle-Ottawa Scale (NOS) for Assessing the Quality of Nonrandomised Studies in Meta-Analyses. https://www.ohri.ca/programs/clinical_epidemiology/oxford.asp.

[44] Ma L.L., Wang Y.Y., Yang Z.H., Huang D., Weng H., Zeng X.T. (2020). Methodological quality (risk of bias) assessment tools for primary and secondary medical studies: What are they and which is better?. Mil. Med. Res..

[45] Stang A. (2010). Critical evaluation of the Newcastle-Ottawa scale for the assessment of the quality of nonrandomized studies in meta-analyses. Eur. J. Epidemiol..

[46] Aromataris E., Lockwood C., Porritt K., Pilla B., Jordan Z. JBI Manual for Evidence Synthesis. https://synthesismanual.jbi.global.

[47] Loh Z.C., Hussain R., Balan S., Saini B., Muneswarao J., Ong S.C., Babar Z.U. (2023). Perceptions, attitudes, and behaviors of asthma patients towards the use of short-acting β2-agonists: A systematic review. PLoS ONE.

[48] Niño de Guzmán E., Martínez García L., González A.I., Heijmans M., Huaringa J., Immonen K., Ninov L., Orrego-Villagrán C., Pérez-Bracchiglione J., Salas-Gama K. (2021). The perspectives of patients and their caregivers on self-management interventions for chronic conditions: A protocol for a mixed-methods overview. F1000Research.

[49] Vallerand R. (2012). From Motivation to Passion: In Search of the Motivational Processes Involved in a Meaningful Life. Can. Psychol.-Psychol. Can..

[50] Baethge C., Goldbeck-Wood S., Mertens S. (2019). SANRA—A scale for the quality assessment of narrative review articles. Res. Integr. Peer Rev..

[51] Sigmundsson H., Dybendal B.H., Loftesnes J.M., Olafsson B., Grassini S. (2022). Passion a key for success: Exploring motivational factors in football players. New Ideas Psychol..

[52] Chamorro J.L., Alcaraz S., Sanchez-Oliva D., Garcia-Calvo T., Torregrossa M. (2021). Fuelling the passion: Psychological needs and behavioural regulations as antecedents of passion for football. J. Sports Sci..

[53] Chamorro J.L., Moreno R., García-Calvo T., Torregrossa M. (2020). The Influence of Basic Psychological Needs and Passion in Promoting Elite Young Football Players’ Development. Front. Psychol..

[54] Chamorro J.L., Torregrosa M., Sánchez Oliva D., Garciá Calvo T., León B. (2016). Future Achievements, Passion and Motivation in the Transition from Junior-to-Senior Sport in Spanish Young Elite Soccer Players. Span. J. Psychol..

[55] Curran T., Appleton P.R., Hill A.P., Hall H.K. (2013). The mediating role of psychological need satisfaction in relationships between types of passion for sport and athlete burnout. J. Sports Sci..

[56] Curran T., Appleton P.R., Hill A.P., Hall H.K. (2011). Passion and burnout in elite junior soccer players: The mediating role of self-determined motivation. Psychol. Sport Exerc..

[57] Hendry D.T., Crocker P.R.E., Hodges N.J. (2014). Practice and play as determinants of self-determined motivation in youth soccer players. J. Sports Sci..

[58] Robazza C., Vitali F., Bortoli L., Ruiz M.C. (2025). Basic psychological needs satisfaction, coping functions, and emotional experiences in competitive athletes: A multi-states theory perspective. Sci. Rep..

[59] Doron J., Hayotte M., d’Arripe-Longueville F., Leprince C. (2023). Coping profiles of adolescent football players and association with interpersonal coping: Do emotional competence and psychological need satisfaction matter?. Scand. J. Med. Sci. Sports.

[60] Yildirim S., Yildiz A., TÜRkerİ Bozkurt H., Bilgin E., Yüksel Y., Koruç Z. (2023). The associations of transformational leadership and team cohesion on the psychological health of young football players through basic psychological needs. Sci. Med. Footb..

[61] Karayel E., Adilogullari I., Senel E. (2024). The role of transformational leadership in the associations between coach-athlete relationship and team resilience: A study on elite football players. BMC Psychol..

[62] Vallerand R.J., Paquette V. (2024). The role of passion in the resilience process. Self Identity.

[63] Rahimi S., Paquette V., Vallerand R.J. (2023). The role of students’ passion and affect in resilience following failure. Learn. Individ. Differ..

[64] Paquette V., Vallerand R.J., Houlfort N., Fredrickson B.L. (2022). Thriving through adversity: The role of passion and emotions in the resilience process. J. Personal..

[65] St-Cyr J., Gavrila A., Tanguay-Sela M., Vallerand R.J. (2024). Perfectionism, disordered eating and well-being in aesthetic sports: The mediating role of passion. Psychol. Sport Exerc..

[66] Woods S., Dunne S., Gallagher P., McNicholl A. (2022). A systematic review of the factors associated with athlete burnout in team sports. Int. Rev. Sport Exerc. Psychol..

[67] Lopes M., Vallerand R.J. (2020). The role of passion, need satisfaction, and conflict in athletes’ perceptions of burnout. Psychol. Sport Exerc..

[68] Nuetzel B. (2023). Coping strategies for handling stress and providing mental health in elite athletes: A systematic review. Front. Sports Act. Living.

[69] de Jonge J., Balk Y.A., Taris T.W. (2020). Mental Recovery and Running-Related Injuries in Recreational Runners: The Moderating Role of Passion for Running. Int. J. Environ. Res. Public Health.

[70] Gu S., Bi S., Guan Z., Fang X., Jiang X. (2022). Relationships among Sports Group Cohesion, Passion, and Mental Toughness in Chinese Team Sports Athletes. Int. J. Environ. Res. Public Health.

[71] Yukhymenko-Lescroart M.A. (2021). The role of passion for sport in college student-athletes’ motivation and effort in academics and athletics. Int. J. Educ. Res. Open.

[72] Verner-Filion J., Vallerand R.J., Amiot C.E., Mocanu I. (2017). The two roads from passion to sport performance and psychological well-being: The mediating role of need satisfaction, deliberate practice, and achievement goals. Psychol. Sport Exerc..

[73] Schellenberg B.J.I., Lötscher J. (2024). Passion and engagement in sport: A look at athletes and coaches using a quadripartite approach. Psychol. Sport Exerc..

[74] Li J., Jiang X., Zhou Y. (2024). Culture, emotion, and cognition: Understanding the psychological dynamics of Chinese sports with emotional regulation skills and cognitive reappraisal. Heliyon.

[75] Bill T., Dessart G., Antonini Philippe R. (2024). Does Ultra-Endurance Passion Make Athletes Happy?. Sports.

[76] Gu S., Peng W., Du F., Fang X., Guan Z., He X., Jiang X. (2023). Association between coach-athlete relationship and athlete engagement in Chinese team sports: The mediating effect of thriving. PLoS ONE.

[77] Huang H., Sha X., Zhong C., Ma N., Shui Y. (2025). Association of football athlete engagement profiles with adolescent mental health —A latent profile analysis. BMC Public Health.

[78] Uğraş S., Mergan B., Çelik T., Hidayat Y., Özman C., Üstün Ü.D. (2024). The relationship between passion and athlete identity in sport: The mediating and moderating role of dedication. BMC Psychol..

[79] Domingues A., Santos J., Batista M., Serrano J., Honório S., Petrica J. (2024). Mediation effects of basic psychological needs and motivation among coach leadership style on the subjective well-being of adapted sports athletes’ members of the special olympics. PLoS ONE.

[80] Huang H., Wang H.H.X., Donaghy E., Henderson D., Mercer S.W. (2022). Using self-determination theory in research and evaluation in primary care. Health Expect..

[81] Wu X., Cai Y., Abidin N.E.Z., Jaladin R.A.M. (2024). Associations between motivational factors and burnout syndrome among elite skiers. BMC Psychol..

[82] Chuented P., Puranitee P., Pakakasama S., Meepanya S. (2023). Factors affecting residents’ internal motivation, grit, and well-being. BMC Med. Educ..

[83] Hong H.J., Wilkinson G., Rocha C.M. (2022). The Relationship Between Basic Needs Satisfaction, Self-determined Motivation, and Burnout in Korean Esports Players. J. Gambl. Stud..

[84] Wu C.-H., Zhao Y.-D., Yin F.-Q., Yi Y., Geng L., Xu X. (2024). Mental Fatigue and Sports Performance of Athletes: Theoretical Explanation, Influencing Factors, and Intervention Methods. Behav. Sci..

[85] González-Hernández J., Gómez-López M., Manzano-Sánchez D., Valero-Valenzuela A. (2023). Motivated and without Fear of Failure: The Strength of Basic Psychological Needs in Youth Spanish Athletes in Team Sports. J. Hum. Kinet..

[86] Mossman L.H., Slemp G.R., Lewis K.J., Colla R.H., O’Halloran P. (2022). Autonomy support in sport and exercise settings: A systematic review and meta-analysis. Int. Rev. Sport Exerc. Psychol..

[87] Brat V., Bota A., Mitrache G., Teodorescu S. (2025). The Motivational Level of Performance Swimmers and Its Impact on the Risk of Sports Dropout. Sports.

[88] Selejó Joó B.T., Czipa H., Bódi R., Lupócz Z., Paronai R., Tóth B.T., Tóth H.L., Kocsner O.C., Lovas B., Lukácsi C. (2024). Qualitative Analysis of Micro-System-Level Factors Determining Sport Persistence. J. Funct. Morphol. Kinesiol..

[89] Nielsen G., Wikman J.M., Appleton P.R., Bentsen P., Elsborg P. (2024). Predicting adolescents’ continuation in club sports: A prospective cohort study of the importance of personal and contextual motivational factors in five sports in Denmark. Scand. J. Med. Sci. Sports.

[90] Bonell Monsonís O., Verhagen E., Spörri J., Gouttebarge V., Bolling C. (2024). ‘Every turn can be the last one I do’—Perceptions of injury risk in high-performance snow sports and its implication for injury prevention. Inj. Prev..

[91] Eliason P.H., Galarneau J.-M., Kolstad A.T., Pankow M.P., West S.W., Bailey S., Miutz L., Black A.M., Broglio S.P., Davis G.A. (2023). Prevention strategies and modifiable risk factors for sport-related concussions and head impacts: A systematic review and meta-analysis. Br. J. Sports Med..

[92] Cardoso-Marinho B., Barbosa A., Bolling C., Marques J.P., Figueiredo P., Brito J. (2022). The perception of injury risk and prevention among football players: A systematic review. Front. Sports Act. Living.

[93] Mühlberger C., Böhm A.M., Hansen J., Behrendt P., Wastian M., Jonas E. (2023). Coaching as a growth- or security-oriented process–How regulatory fit increases coaching success. PLoS ONE.

[94] Debanne T., Volossovitch A. (2022). Team Regulatory Strategies and Performance in Elite Handball. Res. Q. Exerc. Sport.

[95] Zhao Y., Wu Q., Zheng W. (2025). Motivation and physical activity across Chinese adolescents: Based on latent profile analysis. PLoS ONE.

[96] Železnik Mežan L., Škof B., Leskošek B., Cecić Erpič S. (2023). Effects of cooperative learning in youth athletics’ motivational climate, peer relationships and self-concept. Phys. Educ. Sport Pedagog..

